# Re-evaluation of Adrenocorticotropic Hormone and Melanocyte Stimulating Hormone Activation of GPR139 *in Vitro*

**DOI:** 10.3389/fphar.2018.00157

**Published:** 2018-03-02

**Authors:** Diane Nepomuceno, Chester Kuei, Curt Dvorak, Timothy Lovenberg, Changlu Liu, Pascal Bonaventure

**Affiliations:** Janssen Research and Development, LLC, San Diego, CA, United States

**Keywords:** GPR139, tryptophan, phenylalanine, GPCR, ACTH, α-MSH and β-MSH, melanocortin receptor

## Abstract

It is now well established that GPR139, a G-protein coupled receptor exclusively expressed in the brain and pituitary, is activated by the essential amino acids L-tryptophan (L-Trp) and L-phenylalanine (L-Phe) via G_αq_-coupling. The *in vitro* affinity and potency values of L-Trp and L-Phe are within the physiological concentration ranges of L-Trp and L-Phe. A recent paper suggests that adrenocorticotropic hormone (ACTH), α and β melanocyte stimulating hormones (α-MSH and β-MSH) and derivatives α-MSH_1-9_/α-MSH_1-10_ can also activate GPR139 *in vitro*. We tested this hypothesis using guanosine 5′-*O*-(3-[^35^S]thio)-triphosphate binding (GTPγS), calcium mobilization and [^3^H]JNJ-63533054 radioligand binding assays. In the GTPγS binding assay, α-MSH, α-MSH_1-9_/α-MSH_1-10_, and β-MSH had no effect on [^35^S]GTPγS incorporation in cell membranes expressing GPR139 up to 30 μM in contrast to the concentration dependent activation produced by L-Trp, JNJ-63533054, and TC-09311 (two small molecule GPR139 agonists). ACTH slightly decreased the basal level of [^35^S]GTPγS incorporation at 30 μM. In the GPR139 radioligand binding assay, a moderate displacement of [^3^H]JNJ-63533054 binding by ACTH and β-MSH was observed at 30 μM (40 and 30%, respectively); α-MSH, α-MSH_1-9_/α-MSH_1-10_ did not displace any specific binding at 30 μM. In three different host cell lines stably expressing GPR139, α-MSH, and β-MSH did not stimulate calcium mobilization in contrast to L-Trp, JNJ-63533054, and TC-09311. ACTH, α-MSH_1-9_/α-MSH_1-10_ only weakly stimulated calcium mobilization at 30 μM (<50% of EC_100_). We then co-transfected GPR139 with the three melanocortin (MC) receptors (MC3R, MC4R, and MC5R) to test the hypothesis that ACTH, α-MSH, and β-MSH might stimulate calcium mobilization through a MCR/GPR139 interaction. All three MC peptides stimulated calcium response in cells co-transfected with GPR139 and MC3R, MC4R, or MC5R. The MC peptides did not stimulate calcium response in cells expressing MC3R or MC5R alone consistent with the G_s_ signaling transduction pathway of these receptors. In agreement with the previously reported multiple signaling pathways of MC4R, including G_q_ transduction pathway, the MC peptides produced a calcium response in cells expressing MC4R alone. Together, our findings do not support that GPR139 is activated by ACTH, α-MSH, and β-MSH at physiologically relevant concentration but we did unravel an *in vitro* interaction between GPR139 and the MCRs.

## Introduction

GPR139 (or GPRg1) was originally identified by two independent teams and characterized as a novel G_q_-coupled orphan receptor exclusively expressed in the central nervous system (Gloriam et al., 2005; Matsuo et al., 2005). A few years later several groups reported surrogate small molecule agonists for GPR139 (Hu et al., 2009; Shi et al., 2011; Wang et al., 2015; Shehata et al., 2016). Based on these known surrogate agonists, Isberg et al. (2014) and Nøhr et al. (2017a) disclosed a pharmacophore model and proposed that L-tryptophan (L-Trp) and L-phenylalanine (L-Phe) were the putative endogenous ligands for GPR139. At the same time, we provided additional and independent biological and pharmacological evidence to support that L-Trp and L-Phe activate GPR139 (Liu et al., 2015). The EC_50_ values in the 30–300 μM range were consistent with the physiological concentration of L-Trp and L-Phe in tissue. Chromatography of rat serum, rat brain and human serum extract confirmed that L-Trp and L-Phe were the only substances detected capable of activating GPR139. We hypothesized that GPR139 might act as a sensor to detect dynamic changes of L-Trp and L-Phe. We also disclosed JNJ-63533054 a new selective brain penetrant GPR139 agonist (Dvorak et al., 2015). Despite the availability of several GPR139 agonist tool compounds the physiological function of GPR139 remains elusive. Noteworthily, there is a shortage of potent and selective GPR139 antagonist.

A recent publication suggests that *in vitro* GPR139 is also activated by ACTH, α-MSH, β-MSH and shorter fragments of α-MSH (α-MSH_1-9_, α-MSH_1-10_) (Nøhr et al., 2017b). Nøhr et al. (2017b) identified the MC4R, a peptide binding GPCR, as having a similar binding cavity to that observed for GPR139. Based on this observation, they experimentally tested MC peptide ligands acting on MC4R for activity on GPR139 using a CHO cell line stably expressing the human GPR139 receptor. They reported that ACTH, α-MSH, β-MSH, α-MSH_1-9_, α-MSH_1-10_ all activate GPR139 in the sub- to low micromolar range. They found that ACTH, α-MSH, and β-MSH were 100-fold less potent on GPR139 than on MC4R. However, the MC peptides were 20- to 120-fold more potent on the GPR139 receptor than L-Trp and L-Phe. They concluded that the potencies of the ACTH and MSH peptides/derivatives were well above endogenous levels and thus were not likely to be the physiologically relevant agonists but that related peptides could be more potent and thus physiologically active. One limitation of the experimental work of Nøhr et al. (2017b) is the use of a single *in vitro* assay (calcium mobilization) in a single host cell line (CHO cells stably expressing human GPR139).

Here, we attempted to reproduce and extend the experimental data reported by Nøhr et al. (2017b) by using several assays ([^35^S]GTPγS binding, calcium mobilization, and [^3^H]JNJ-63533054 radioligand binding assays) and multiple host cell lines (CHO-TREx, HEK-293, SK-N-MC/CRE-TREx). The MC peptides did not activate [^35^S]GTPγS activity nor displace [^3^H]JNJ-63533054 binding to cell membranes expressing GPR139 up to 30 μM except for ACTH and β-MSH. A weak inverse agonist effect was observed for ACTH in the GTPγS binding assay and a small displacement was observed at 30 μM for ACTH and β-MSH in the radioligand binding assay. In three different host cell lines stably expressing GPR139, α-MSH, and β-MSH did not stimulate calcium mobilization in contrast to L-Trp, JNJ-63533054, and TC-09311. ACTH, α-MSH_1-9_/α-MSH_1-10_ only weakly stimulated calcium mobilization at 30 μM (<50% of maximal response). Therefore, we explored a potential interaction between GPR139 and the MCRs by performing co-transfection experiments. We observed that ACTH, α-MSH, and β-MSH stimulated a concentration dependent calcium response in cells co-transfected with GPR139 and MC3R, MC4R or MC5R. In the absence of GPR139 expression, ACTH, α-MSH, and β-MSH did not stimulate a calcium response in cells exclusively expressing MC3R or MC5R consistent with the G_s_-signaling transduction pathway for MCRs. In agreement with the previously reported G_q_ transduction pathway of MC4Rs, ACTH, α-MSH, and β-MSH produced a concentration dependent calcium response in cells expressing MC4R alone. The findings from the present study do not support that GPR139 is activated by ACTH, α-MSH, and β-MSH at physiologically relevant concentration but unravel an *in vitro* interaction between GPR139 and the MCRs which will warrant further investigations.

## Materials and Methods

### Chemicals

The following peptides and compounds were purchased: ACTH (1–39), human (Tocris, Oxford, United Kingdom, #3492, batch 7), α-MSH (Tocris, Oxford, United Kingdom, #2584, batch 4), β-MSH (Bachem, Torrance, CA, United States, #H-1475, batch 2500965), α-MSH_1-9_/α-MSH_1-10_ (InnoPep, San Diego, CA, United States, Synthesis ID F082237/F082236), TC-O 9311 disclosed as Compound 1a by Shi et al. (2011) (Tocris, Oxford, United Kingdom, #4255, batch 1), L-Trp (Sigma, St. Louis, MO, United States, #T0254, batch 080M02471V), HS-024 (Tocris, Oxford, United Kingdom, #1832, batch 4). JNJ-63533054 (3-chloro-*N*-[2-oxo-2-[[(1S)-1-phenylethyl]amino]ethyl]benzamide) and JNJ-3792165 (1H-pyrazole-3-carboxamide, 1-[(2,6-dichlorophenyl)methyl]-5-methyl-*N*-(3-methylphenyl)) were synthesized in-house at Janssen Research and Development, LLC. (San Diego, CA, United States) (Dvorak et al., 2015). JNJ-63533054 was tritiated at Moravek Biochemicals (Brea, CA, United States).

Peptides (ACTH, α-MSH, α-MSH_1-9_, α-MSH_1-10_, and β-MSH) and L-Trp were solubilized in water ([^35^S]-GTPγS and receptor binding assays) or HBSS (calcium mobilization assays) using Eppendorf Protein Lobind tubes (Sigma, St. Louis, MO, United States, #Z666513). TC-09311, JNJ-63533054, and JNJ-3792165 were solubilized in DMSO. Serial dilutions ranging from 30 μM–1.9 nM for ACTH, α-MSH, and β-MSH, 10 μM–0.64 nM for JNJ-63533054 and TC-O9311, 3 μM–192 nM for L-Trp were made in their respective assay buffer.

Pertussis toxin (specific inhibitor of G_i/o_) was purchased from Tocris (Oxford, United Kingdom, #3097, batch 13) and YM-254890 (specific inhibitor of G_q_) was purchased from AdipoGen (San Diego, CA, United States, #AG-CN2-0509).

### Recombinant and Stable Expression of GPR139 and MCR

Human MC3R (NM_019888), MC4R (NM_005912), and MC5R (NM_005913) were transiently expressed in HEK-293 cells with and without human GPR139 (KR081941) or the chimeric G-protein Gα_16_. All genes were cloned into pCIneo (Promega, Madison, WI, United States, #E1841). HEK-293 cells were cultured in DMEM/F12 (Gibco, Grand Island, NY, United States, #11039-021), 10% FBS (Hyclone, Logan, UT, United States, # SH300700.03), 50 units/ml penicillin and 50 μg/ml streptomycin (Mediatech, Manassas, VA, United States, #30-001-CI). Cells were seeded at 4 × 10^6^ cells in 10 cm dishes and allowed to grow overnight at 37°C, 5% CO_2_. On the day of transfection, culture media was replaced with F12-K (Mediatech, Manassas, VA, United States, #10-025-CV), 10% FBS, 20 mM HEPES (Hyclone, Logan, UT, United States, #SH30237.01) and transfected with 15 μg DNA (10 μg MCR:5 μg pCIneo, 10 μg MCR:5 μg Gα_16_ or 7.5 μg MCR:7.5 μg GPR139) using FuGene HD (Promega, Madison, WI, United States, #E2311) according to manufacturer’s instructions. MC3R, MC4R, and MC5R were also transiently expressed in the following GPR139 stably expressing cell lines using the FuGENE HD method: HEK-293 GPR139 clone#33, CHO-TREx GPR139 clone#18, and SK-N-MC/CRE TREx GPR139 clone#35. Stable cell lines were established according to method previously described (Liu et al., 2015).

### [^35^S]-GTPγS Binding Assay

Membranes prepared from CHO-TRex cells stably expressing GPR139 were incubated with several concentrations of test compounds ranging from 1.9 nM–30 μM (ACTH, α-MSH, α-MSH_1-9,_ α-MSH_1-10,_ and β-MSH), 0.13 nM–10 μM (JNJ-63533054 and TC-O9311) and 38 nM–3 mM (L-Trp) and 50 pM [^35^S]GTPγS (Perkin-Elmer, Waltham, MA, United States) in assay buffer (50 mM Tris-HCl, pH 7.4, 100 mM NaCl, 10 mM MgCl_2_, 1 mM EDTA, 10 μM GDP) for 1 h at room temperature. For G-protein inhibitor experiments, CHO-TRex cells stably expressing GPR139 were treated with 100 ng/ml pertussis toxin (G_i/o_ inhibitor) overnight prior to harvesting cells for membrane preparations. The G_q_ inhibitor, YM-254890 (1 μm) was added to membrane preparation prior to incubation with test compounds and [^35^S]GTPγS. Reactions were terminated by rapid filtration through GF/C filterplates (Perkin-Elmer, Waltham, MA, United States) on a Cell Harvester (Perkin-Elmer, Waltham, MA, United States). Filterplates were washed three times with ice-cold wash buffer (50 mM Tris, 5 mM MgCl_2_) and Microscint-40 was added to each well. Bound radioactivity was measured on a Topcount scintillation counter (Perkin-Elmer, Waltham, MA, United States). Data was normalized to background signal and plotted as % stimulation on GraphPad Prism 7.0, 100% stimulation was the maximal response (EC_100_) observed with JNJ-63533054. EC_50s_ were calculated by modeling the response associated with the logarithm of concentration as a three-parameter logistic curve with the Bottom plateau parameter fixed at 0. The fit was obtained using least-squares non-linear regression as implemented in GraphPad Prism 7.0.

### Radioligand Binding Assay

Membranes prepared from CHO-TRex cells stably expressing human GPR139 were incubated with several concentrations of test compounds ranging from 1.9 nM–30 μM (ACTH, α-MSH, α-MSH_1-9,_ α-MSH_1-10_, and β-MSH), 0.13 nM–10 μM (JNJ-63533054 and TC-O9311) and 38 nM–3 mM (L-Trp) and 10 nM [^3^H] JNJ-63533054 (24.7 Ci/mmol) for 1 h at room temperature. Reaction was terminated by filtration through GF/C filterplates pre-treated with PEI (polyethyleneimine) followed by washes with ice-cold TE buffer. Filterplates were dried in a 60°C oven followed by addition of Microscint-0. Bound radioactivity was read on a Topcount scintillation counter. Non-specific binding was determined with 10 μM TC-0 9311. Half-maximal inhibitory concentrations (IC_50s_) of test compounds were calculated by fitting a three-parameter logistic curve using least-squares non-linear regression analysis as implemented in Graphpad Prism 7.0. The Top plateau was fixed at 100 and the Bottom was fixed at 0. K_i_’s were determined according to the Cheng-Prussoff equation using the K_d_ (10 nM) previously described (Liu et al., 2015). IC_50_ values from incomplete curves were calculated by constraining the bottom of the curve to the binding obtained in the presence of 10 μM TC-O9311.

### Calcium Mobilization Assay

Intracellular calcium mobilization was measured with the BD calcium kit (BD Bioscience, San Jose, CA, United States). The recombinant cell lines expressing MC3R, MC4R, and MC5R with and without GPR139 or Gα_16_ were seeded in culture media at a density of 50,000 cells/well into 96-well black/clear-bottom poly-D-lysine treated tissue culture plates. Cells were incubated overnight at 37°C and 5% CO_2_. Next day, culture media was removed and cells were washed with HBSS prior to dye-loading for 1 h at 37°C and 5% CO_2_. Agonist stimulation and intracellular calcium measurements were done on the FLIPR Tetra (Molecular Devices, Sunnyvale, CA, United States). For receptor inhibition analysis, antagonists (1 μM HS-024 or 10 μM JNJ-3792165) were added off-line 15 min prior to agonist addition on the Tetra. For G-protein inhibitor experiments, cells were treated with 1 μM YM-254890 for 5 min prior to agonist additions on the Tetra. Responses were normalized to buffer (0%) and EC_100_ of JNJ-63533054 (100%) in **Figures 3A–C**, **4C,F,I**. In **Figures 4A,B,D,E,G,H** responses were normalized to EC_100_ of ACTH. Responses in **Figure 6** were normalized to its respective agonist response without antagonist. EC_50s_ were calculated by fitting a three-parameter logistic curve using least-squares non-linear regression analysis as implemented in Graphpad Prism 7.0.

## Results

### ACTH, α-MSH, and β-MSH Do Not Stimulate [^35^S]-GTPγS Incorporation in CHO-TREx Cells Stably Expressing Human GPR139

**FIGURE 1:**
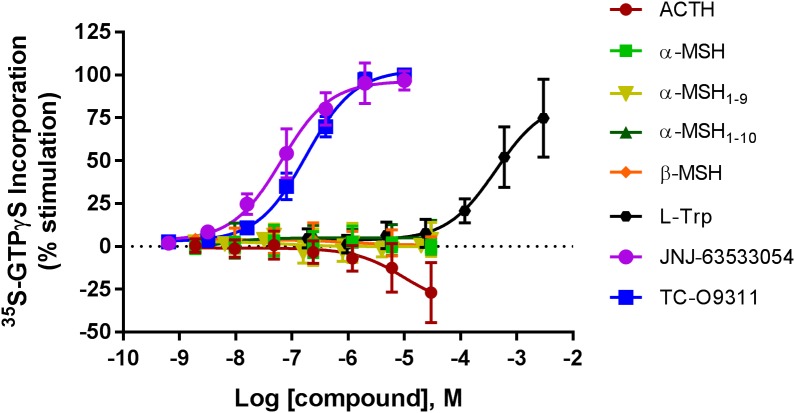
Concentration response curves of ACTH, α-MSH, α-MSH_1-9_, α-MSH_1-10_, β-MSH, L-Trp, JNJ-63533054, and TC-O9311 on [^35^S]GTPγS binding in CHO-TREx cells stably expressing human GPR139. Data are the mean ± SEM of at least three separate experiments performed in triplicates. Response normalized to buffer and EC_100_ of JNJ-63533054.

To determine if the MC agonists ACTH, α-MSH, α-MSH derivatives (α-MSH_1-9_, α-MSH_1-10_) and β-MSH activate GPR139, we measured [^35^S]-GTPγS incorporation, the first step in activation of GPCRs, in CHO-TREx cells stably expressing GPR139 (**Figure 1**). α-MSH and β-MSH did not activate GPR139 up to 30 μM as shown by the lack of [^35^S]-GTPγS incorporation. In addition, the α-MSH derivatives α-MSH_1-9_ and α-MSH_1-10_ which were reported by Nøhr et al. (2017b) to be 5- to 10-fold more potent than ACTH, also failed to activate GPR139 in this assay. Interestingly, ACTH slightly decreased basal [^35^S]-GTPγS incorporation 25% below basal levels. The inability to stimulate [^35^S]-GTPγS incorporation by the peptides was not due to lack of GPR139 expression or function because the GPR139 agonists L-Trp (pEC_50_ = 3.37 ± 0.16), JNJ-63533054 (pEC_50_ = 7.19 ± 0.08), and TC-O 9311 (pEC_50_ = 6.75 ± 0.04) produced a robust concentration-dependent increase of [^35^S]-GTPγS binding. We then investigated if the GTPγS assay reflects G_i/o_ or G_q_ signaling by using the specific inhibitors pertussis toxin (G_i/o)_ and YM-254890 (G_q_). After pertussis toxin treatment, TC-O9311, JNJ-6353354, and L-Trp did not stimulate [^35^S]-GTPγS incorporation demonstrating that the response is mediated through G_i_ (data not shown). YM-254890 had no effect on TC-O9311, JNJ-63533054 and L-Trp response ruling out an involvement of G_q_ (data not shown).

### α-MSH, α-MSH_1-9_, and α-MSH_1-10_ Do Not Compete With [^3^H]JNJ-63533054 Binding in CHO-TREx Cells Stably Expressing Human GPR139. ACTH and β-MSH Partially Compete With [^3^H]JNJ-63533054 Binding at High Concentration

**FIGURE 2:**
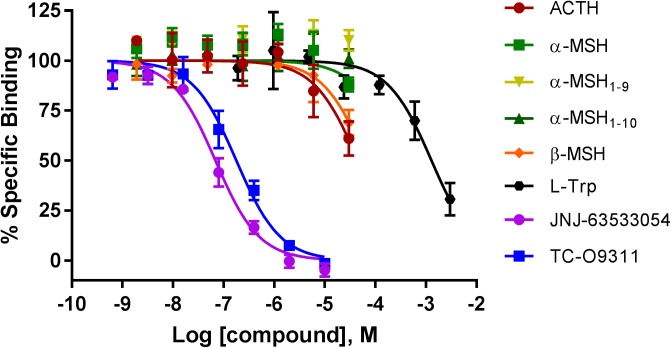
Competition of [^3^H]-JNJ63533054 binding by MC ligands ACTH, α-MSH, α-MSH_1-9_, α-MSH_1-10_, β-MSH, and GPR139 ligands L-Trp, JNJ-63533054, and TC-O9311 in CHO-TREx cells stably expressing human GPR139. Data are the mean ± SEM of at least three separate experiments performed in triplicates.

We next investigated if ACTH, α-MSH, α-MSH_1-9_, α-MSH_1-10_, and β-MSH compete for [^3^H]JNJ-63533054 binding in CHO-TREx cell stably expressing human GPR139 (**Figure 2**). We had previously demonstrated that [^3^H]JNJ-63533054 bound to cell membranes expressing GPR139 and could be specifically displaced by L-Trp and L-Phe (Liu et al., 2015).

Similar to the results obtained with the [^35^S]GTPγS assay, α-MSH and its derivatives, α-MSH_1-9_ and α-MSH_1-10_ did not compete with [^3^H]JNJ-63533054 (**Figure 2**). However, ACTH and β-MSH partially displaced [^3^H]-JNJ-63533054 binding at the highest concentration tested (40% and 30% at 30 μM, respectively). The positive controls L-Trp (pK_i_ = 3.19 ± 0.08), JNJ-63533054 (pK_i_ = 7.46 ± 0.04), and TC-O 9311 (pK_i_ = 7.05 ± 0.05) displaced [^3^H]-JNJ63533054 binding in a concentration-dependent manner.

### α-MSH and β-MSH Do Not Induce Calcium Mobilization in Three Recombinant Host Cells Stably Expressing Human GPR139. A Weak Activation Was Observed at High Concentration for ACTH and α-MSH Derivatives

**FIGURE 3:**
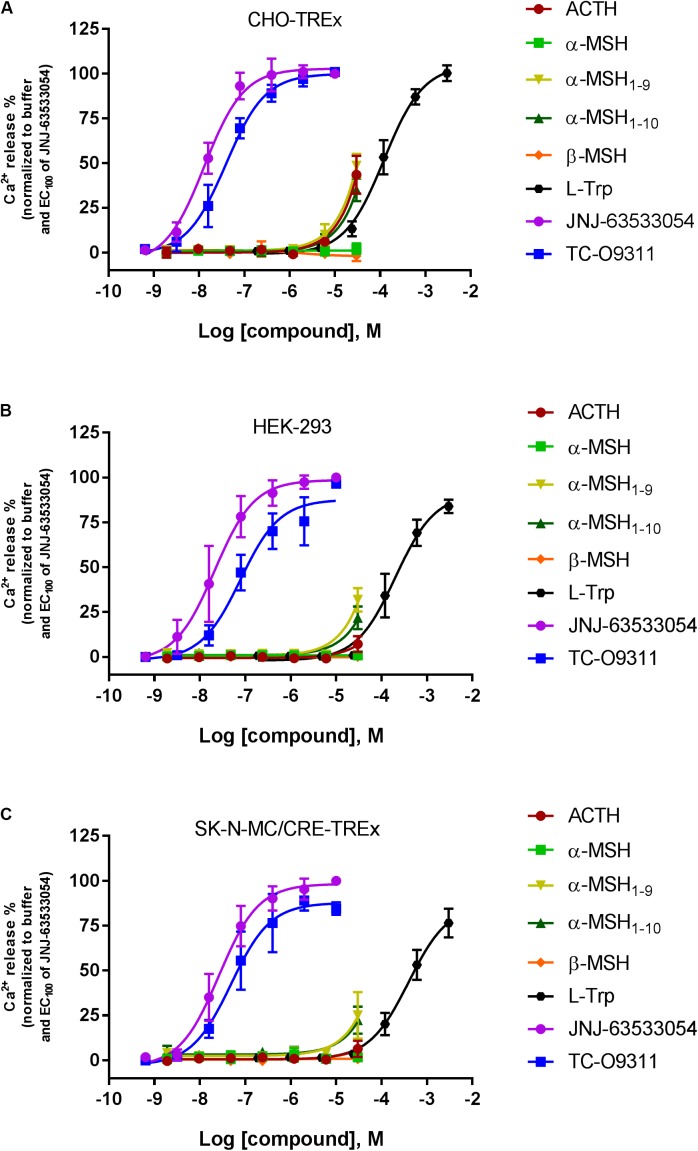
Concentration response of the MC agonists ACTH, α-MSH, β-MSH, and GPR139 agonists (L-Trp, JNJ-63533054, and TC-O9311) on calcium mobilization in three different host cells stably expressing human GPR139: **(A)** CHO-TREx, **(B)** HEK-293, and **(C)** SK-N-MC/CRE-TREx. Data are the mean ± SEM of at least three separate experiments performed in duplicates.

Stable expression of GPR139 was established in three different host cells: CHO-TREx (**Figure 3A**), HEK-293 (**Figure 3B**), and SK-N-MC/CRE (**Figure 3C**). GPR139 cell lines, CHO-TREx and SK-N-MC/CRE, were treated with 10 μM doxycycline to induce expression of GPR139. In the CHO-TRex stable line (**Figure 3A**), ACTH and the small peptide fragments α-MSH_1-9_ and α-MSH_1-10_ weakly activated GPR139 at the highest concentration tested (44%, 48%, and 35% at 30 μM, respectively). In the HEK-293 and SK-N-MC/CRE stable lines, weak activation was also observed with α-MSH_1-9_ and α-MSH_1-10_ (HEK-293: 32% and 22%, respectively; SK-N-MC/CRE: 25% and 22%, respectively). No activity was detected with α-MSH and β-MSH in all three stable cell lines. GPR139 control agonists all produced a concentration-dependent increase in intracellular calcium (CHO-TREx; L-Trp pEC_50_ = 3.91 ± 0.05, JNJ-63533054 pEC_50_ = 7.88 ± 0.05 and TC-O 9311 pEC_50_ = 7.40 ± 0.06; HEK-293: L-Trp pEC_50_ = 3.69 ± 0.06, JNJ-63533054 pEC_50_ = 7.67 ± 0.08 and TC-O 9311 pEC_50_ = 7.11 ± 0.13; SK-N-MC/CRE: L-Trp pEC_50_ = 3.40 ± 0.06, JNJ-63533054 pEC_50_ = 7.58 ± 0.07 and TC-O 9311 pEC_50_ = 7.31 ± 0.12) (**Figure 3**).

### ACTH, α-MSH, and β-MSH Induced Calcium Mobilization in HEK-293 Cells Transiently Co-expressing Human GPR139 With MC3R, MC4R, and MC5R

**FIGURE 4:**
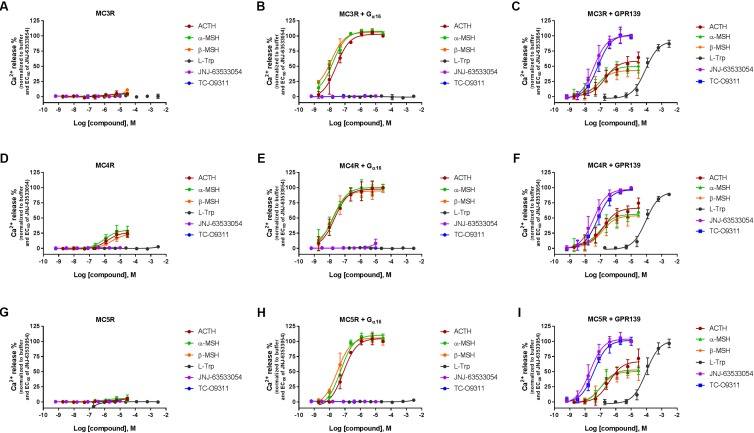
Concentration response of the MC agonists ACTH, α-MSH, β-MSH, and GPR139 agonists (L-Trp, JNJ-63533054, and TC-O9311) on calcium mobilization in HEK-293 cells transiently expressing MC3R **(A)**, MC3R and G_α16_ **(B)**, MC3R and GPR139 **(C)** MC4R **(D)**, MC4R and G_α16_ **(E)** MC4R and GPR139 **(F)** and MC5R **(G)**, MC5R and G_α16_ **(H)** MC5R and GPR139 **(I)**. Data are the mean ± SEM of at least three separate experiments performed in duplicates.

To investigate a potential interaction between GPR139 and the MCRs, we transiently co-expressed MC3R, MC4R, and MC5R with and without the chimeric G-protein, G_α16_, and GPR139 (**Figure 4**). As expected, ACTH, α-MSH, and β-MSH did not produce a calcium response in MC3R or MC5R expressing cells consistent with the reported G_s_-coupled signaling transduction pathway of MCRs (**Figures 4A,G**). In contrast, ACTH, α-MSH, and β-MSH stimulated calcium release in a concentration-dependent manner in MC4R expressing cells (pEC_50_ = 5.86 ± 0.17, 6.03 ± 0.22, 5.56 ± 0.15 μM, respectively) (**Figure 4D**). However, the respective E_max_ were 27% (ACTH), 30% (α-MSH), and 24% (β-MSH). This is in agreement with published literature reporting multiple signaling pathways for MC4R (Breit et al., 2011) including the G_q_ pathway. However, when MC3R, MC4R, or MC5R are co-expressed with G_α16,_ a chimeric G-protein capable of forcing signaling transduction pathway through G_q_ (intracellular calcium release), ACTH, α-MSH, and β-MSH all produced a concentration-dependent increase in intracellular calcium (**Figures 4B,E,H**) with the pEC_50s_ in the low nM range as listed in **Table 1**. No calcium response was observed with the GPR139 agonists. Interestingly, when we co-expressed MC3R, MC4R, and MC5R with GPR139 (without G_α16_), ACTH, α-MSH, and β-MSH retained the ability to stimulate intracellular calcium release in a concentration-dependent manner indicating an interaction of these MCRs with GPR139 (**Figures 4C,F,I**). The maximal level of stimulation was about 50% of the response observed with the GPR139 agonists. The potency values were shifted to the right compared to the values measured in cells transiently expressing the MCRs and G_α16_ (pEC_50s_ listed in **Table 1**).

**Table 1:** Agonist potency of MC peptides and GPR139 agonists on HEK-293 transiently expressing MC3R, MC4R, and MC5R with and without G_α16_ or GPR139.

	MC3R	MC3R + G_α16_	MC3R + GPR139	MC4R	MC4R + G_α16_	MC4R + GPR139	MC5R	MC5R + G_α16_	MC5R + GPR139
ACTH	<5.5	7.56 ± 0.13	6.68 ± 0.14	5.86 ± 0.17	7.67 ± 0.15	6.80 ± 0.14	<5.5	7.11 ± 0.08	6.43 ± 0.13
α-MSH	<5.5	7.78 ± 0.11	6.93 ± 0.21	6.03 ± 0.22	7.71 ± 0.16	6.97 ± 0.20	<5.5	7.28 ± 0.09	6.96 ± 0.13
β-MSH	<5.5	7.89 ± 0.09	7.15 ± 0.24	5.56 ± 0.15	7.84 ± 0.15	6.90 ± 0.21	<5.5	7.50 ± 0.10	6.97 ± 0.20
JNJ-63533054	<5.0	<5.0	7.43 ± 0.09	<5.0	<5.0	7.45 ± 0.09	<5.0	<5.0	7.68 ± 0.08
TC-O9311	<5.0	<5.0	7.16 ± 0.08	<5.0	<5.0	7.18 ± 0.08	<5.0	<5.0	7.43 ± 0.06
L-Trp	<2.5	<2.5	4.06 ± 0.06	<2.5	<2.5	4.03 ± 0.05	<2.5	<2.5	4.02 ± 0.07

*pEC_50_ values represent mean ± SEM (*n* = 2–4)*.

**FIGURE 5:**
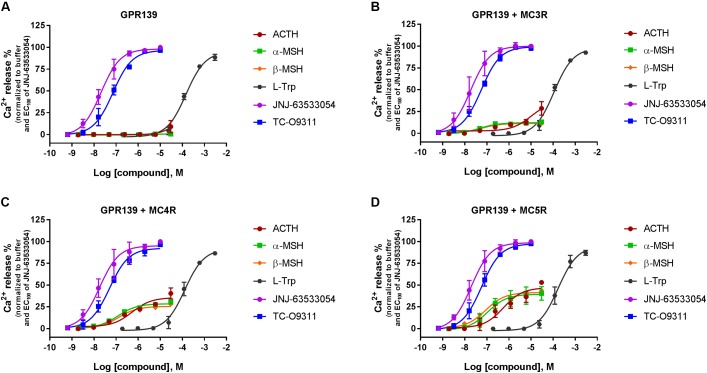
Concentration response of the melanocortin agonists ACTH, α-MSH, β-MSH, and GPR139 agonists (L-Trp, JNJ-63533054, and TC-O9311) calcium mobilization in a stable HEK-293 cell line expressing GPR139 **(A)** transiently co-expressing MC3R **(B)**, MC4R **(C)**, or MC5R **(D)**. Data are the mean ± SEM of at least three separate experiments performed in duplicates.

We then transiently expressed MC3R, MC4R, or MC5R in HEK-293 cells stably expressing GPR139 (**Figure 5**). In agreement with our previous data, ACTH, α-MSH, and β-MSH did not increase calcium mobilization in HEK-293 cells exclusively expressing GPR139 (**Figure 5A**). In the GPR139 stable cells transiently co-expressing MC4R and MC5R the peptides produced a concentration-dependent increase in calcium release (**Figures 5B–D**). Potency values (pEC_50s_ listed in **Table 2**) were consistent with the results obtained in the MCR-GPR139 transient co-transfection experiments in HEK-293 cells (**Table 1**). The response to all three MC peptides in the stable GPR139 cell line transiently expressing MC3R was significantly weaker (<20% stimulation). These data provide further evidence to support an *in vitro* interaction between MCRs and GPR139.

**Table 2:** Agonist potency of MC peptides and GPR139 agonists on a stable GPR139-HEK-293 cell line transiently expressing MC3R, MC4R, and MC5R.

	MC3R	MC4R	MC5R
ACTH	<25% 30 μM	6.44 ± 0.16	6.37 ± 0.15
α-MSH	<25% 30 μM	6.97 ± 0.13	7.00 ± 0.14
β-MSH	<25% 30 μM	6.65 ± 0.20	7.22 ± 0.19
JNJ-63533054	7.69 ± 0.08	7.76 ± 0.08	7.80 ± 0.07
TC-O9311	7.30 ± 0.04	7.21 ± 0.06	7.38 ± 0.07
L-Trp	3.90 ± 0.06	3.86 ± 0.06	3.79 ± 0.06

*pEC_*50*_ values represent mean ± SEM (*n* = 2–3)*.

### ACTH, α-MSH, and β-MSH Induced Calcium Mobilization in Cells Co-expressing MCRs and GPR139 Is Suppressed by a MCR Antagonist (HS-024) But Not by a GPR139 Antagonist (JNJ-3792165)

**FIGURE 6:**
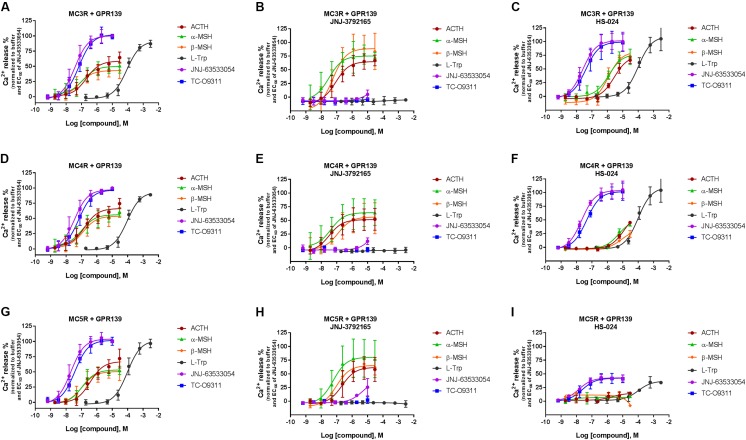
Inhibition of ACTH, α-MSH, β-MSH, L-Trp, JNJ-63533054, or TC-O9311 induced calcium release in HEK-293 cells transiently co-expressing GPR139 and MC3R **(A–C)**, MC4R **(D–F)** or MC5R **(G–I)** by a GPR139 antagonist (10 μM JNJ-3792165, **B, E, H**) or a MCR antagonist (1 μM HS-024, **C, F, I**). Data are the mean ± SEM of at least three separate experiments performed in duplicates.

To further characterize the ACTH, α-MSH, and β-MSH induced calcium response in cells co-expressing MCRs-GPR139 we studied the effect of a MCR antagonist (1 μM HS-024) or a GPR139 antagonist (10 μM JNJ-3792165). Data are presented in **Figure 6**.

JNJ-3792165 has high affinity for human GPR139 (pK_i_ = 7.7, displacement of [^3^H] JNJ-6353054). It behaves as an antagonist in the [^35^S]GTPγS assay (pK_b_ = 7.4) and calcium mobilization assay (pK_b_ = 6.9). JNJ-3792165 was screened for selectivity in a panel of over 50 other neurotransmitter and neuropeptide receptors including the MCRs and had no significant affinity for any receptor (less than 50% inhibition at 1 μM) other than GPR139.

In agreement with the data reported in **Figures 4** and **5**, the peptides produced a concentration-dependent increase in calcium release in cells co-expressing MCRs and GPR139 (**Figures 6A,D,G**). JNJ-3792165 fully inhibited the calcium response to the GPR139 agonists (JNJ-63533054, TC-09311, and L-Trp) but did not block the response induced by ACTH, α-MSH, and β-MSH on all three MCRs (**Figures 6B,E,H**).

The MCR antagonist HS-024 fully blocked the ACTH, α-MSH, and β-MSH induced calcium response in cells co-expressing GPR139 and MC5R (**Figure 6I** and **Table 3**). Interestingly, HS-024 reduced the efficacy of GPR139 agonists (JNJ-63533054, TC-09311, and L-Trp) without decreasing the potency. In cells co-expressing GPR139 and MC3R, HS-024 produced a rightward shift of the EC_50_ for ACTH, α-MSH, and β-MSH without a reduction in efficacy (**Figure 6C** and **Table 3**). In cells co-expressing GPR139 and MC4R, HS-024 produced a reduction in both potency and efficacy for ACTH, α-MSH, and β-MSH (**Figure 6F** and **Table 3**). HS-024 had no effect on the GPR139 agonists in both GPR139-MC3R and MC4R co-expressing cells.

**Table 3:** Agonist potency of MC peptides and GPR139 agonists in the presence of a GPR139 antagonist (10 μM JNJ-3792165) or melanocortin antagonist (1 μM HS-024) in HEK-293 cells transiently expressing MC3R, MC4R, or MC5R and GPR139.

	MC3R + GPR139			MC4R + GPR139			MC5R + GPR139		
	
	No antagonist	JNJ-3792165	HS-024	No antagonist	JNJ-3792165	HS-024	No antagonist	JNJ-3792165	HS-024
ACTH	6.68 ± 0.14	7.06 ± 0.20	5.55 ± 0.10	6.80 ± 0.14	7.49 ± 0.31	4.91 ± 0.08	6.43 ± 0.13	6.66 ± 0.26	>4.5
α-MSH	6.93 ± 0.21	7.50 ± 0.31	5.91 ± 0.16	6.97 ± 0.20	7.38 ± 0.42	5.27 ± 0.11	6.96 ± 0.13	7.23 ± 0.35	>4.5
β-MSH	7.15 ± 0.24	7.26 ± 0.26	5.93 ± 0.14	6.9 ± 0.21	6.91 ± 0.39	5.08 ± 0.14	6.97 ± 0.20	7.02 ± 0.15	>4.5
JNJ-63533054	7.43 ± 0.09	>5	7.60 ± 0.13	7.45 ± 0.09	>5	7.66 ± 0.10	7.68 ± 0.08	>5	7.85 ± 0.13
TC-O9311	7.16 ± 0.08	>5	7.40 ± 0.16	7.18 ± 0.08	>5	7.34 ± 0.09	7.43 ± 0.06	>5	7.63 ± 0.22
L-Trp	4.06 ± 0.06	>2.5	4.00 ± 0.11	4.03 ± 0.05	>2.5	4.00 ± 0.11	4.02 ± 0.07	>2.5	4.00 ± 0.22

*pEC_*50*_ values represent mean ± SEM (*n* = 2–3)*.

## Discussion

The physiological function of GPR139 remains elusive despite its identification more than a decade ago by Matsuo et al. (2005). GPR139 is expressed exclusively in the CNS and pituitary. In particular, high expression of GPR139 has been reported in the medial habenula and lateral septum (Liu et al., 2015). The habenula is part of the limbic circuits involved in the regulation of mood and stress. Neurons from the lateral septum have been shown to modulate neuroendocrine and behavioral stress response (Singewald et al., 2011). The expression of GPR139 in the paraventricular nucleus, arcuate hypothalamus and pituitary suggests it may play a role in the regulation of stress, hormone production/release, growth and metabolism. Recently, Nøhr et al. (2017b) identified the MC4R, a peptide binding GPCR, as having a similar binding cavity to that observed for GPR139. Indeed, all class A GPCRs share high similarities. Sequence and structure analyses show that several GPCRs have higher similarities to MC4R than GPR139 including LPAR1, LPAR2, LPAR3, S1P1, S1P2. However, there is no experimental evidence that these GPCRs interact with MC peptides. Nonetheless, based on this observation, Nøhr et al. (2017b) experimentally tested the activity of the MC peptides and their derivatives for GPR139 in a CHO cell line stably expressing the human GPR139. They reported that ACTH, α-MSH, α-MSH_1-9_, α-MSH_1-10_, and β-MSH all activate GPR139 in the sub- to low micromolar range. Here, we tested this hypothesis in a variety of host cell lines and assay formats. First, we tested the activity of the MC peptides in a [^35^S]GTPγS assay, the first step in activation of GPCRs, in CHO-TREx cells stably transfected with the human GPR139. In contrast to the positive controls (L-Trp, JNJ-63533054, and TC-09311), the MC peptides did not activate GPR139 in this assay. Interestingly, ACTH produced a slight decrease in basal levels of [^35^S]GTPγS incorporation at the highest concentration tested (30 μM). GPR139 displays high constitutive activity when expressed in recombinant systems (Matsuo et al., 2005; Liu et al., 2015) thus ACTH could be acting as a weak inverse agonist on GPR139. We then demonstrated that the GPR139 mediated response of L-Trp, JNJ-63533054 and TC-09311 is sensitive to pertussis toxin, a specific inhibitor of G_i,_ but not to YM-254890 an inhibitor of G_q_ response. This could explain why we were able to measure a response without the expression of a chimeric G_o2_ protein like we had previously reported (Liu et al., 2015). The signaling pathway of GPR139 in our stable GPR139 cell line agrees with previously published reports of GPR139 coupling to an inhibitory G-protein (Süsens et al., 2006). Interestingly, despite evidence of G_i_ signaling in the GTPγS assay we were unable to detect a response for L-Trp, JNJ-63533054, and TC-09311 in a cAMP assay (unpublished data). This is in agreement with a recent study by Shehata et al. (2016) where JNJ-63533054 or compound 1a (TC-O9311) were unable to induce or inhibit a cAMP response. Based on these results, we concluded that the MC peptides, with the exception of ACTH at high concentration, do not activate the GPR139/G_i_ signal transduction pathway. The G_i_ mediated response observed with our GPR139 agonists could explain why α/β-MSH and the α-MSH derivatives α-MSH_1-9_ and α-MSH_1-10_ did not activate GPR139 in this assay, α/β-MSH and the α-MSH derivatives α-MSH_1-9_ and α-MSH_1-10_ may be biased toward the G_q_ signaling pathway.

We then used a GPR139 radioligand binding assay to determine the MC peptide affinities to GPR139. Our results showed that the peptides α-MSH and its derivatives α-MSH_1-9_ and α-MSH_1-10_ did not compete with [^3^H]JNJ-63533054, a selective tracer for GPR139 in CHO-TREx cells stably expressing GPR139. L-Trp, JNJ-63533054, and TC-09311, as previously reported displaced selective [^3^H]JNJ-63533054 binding. ACTH and β-MSH partially competed with [^3^H]JNJ-63533054 at 30 μM. The relatively large peptides might not fully share overlapping binding sites with [^3^H]JNJ-63533054, thus lack of binding competition does not prove lack of MC peptide binding to GPR139. Noteworthy, we were unable to detect [^3^H]JNJ-63533054 binding in a native system, both in rat brain homogenates and brain slices using autoradiography (unpublished data).

Because we were unable to confirm activity of the MC peptides to GPR139 in both the GTPγS and radioligand binding assays, we tested the peptides in a calcium mobilization assay, the assay used by Nøhr et al. (2017b). We ran the calcium mobilization assay in three recombinant host cells (CHO-TREx, HEK-293, and SK-N-MC/CRE) stably expressing human GPR139. In all three different host cell lines expressing GPR139, α-MSH, and β-MSH did not stimulate calcium mobilization in contrast to L-Trp, JNJ-63533054, and TC-09311. However, we could detect a very weak partial stimulation for ACTH, α-MSH_1-9_/α-MSH_1-10_ at 30 μM in all three stably expressing cell lines with the following rank order of potency: CHO-TREx > HEK-293 > SK-N-MC/CRE. This difference in potency between the three different host cells could be attributable to the expression levels of GPR139. Noteworthy, ACTH consistently displayed weak activity in all three assay formats ([^35^S]GTPγS, receptor binding and calcium mobilization). However, potency and efficacy values of ACTH were much weaker than the values reported by Nøhr et al. (2017b).

The lack or weak activities of the MC peptides observed in different assays using multiple GPR139 expressing cell lines led us to postulate that GPR139 might interact with MCRs to switch their signal transduction pathway from a G_s_- to a G_q_. Our data from the co-transfection experiments indicated that ACTH, α-MSH, and β-MSH were not able to induce a calcium response in cells transfected with the MC3R or MC5R alone, consistent with the G_s_-coupled transduction pathway of these receptors (**Figures 4A,G**). MC4Rs, on the other hand, have been reported to couple to both G_q_ and G_s_ (Mountjoy et al., 2001; Simon et al., 2009). In addition, the MC4R-mediated calcium signaling has been shown to be dependent on the cellular context (Newman et al., 2006; Buch et al., 2009). In agreement with the literature, we observed an ACTH, α-MSH, and β-MSH induced calcium signal in cells transfected with MC4R alone (**Figure 4D**). Furthermore, when MCRs were co-transfected with the chimeric G _α16_ protein to force the signal transduction toward the calcium pathway, ACTH, α-MSH, and β-MSH produced a robust concentration dependent calcium response (**Figures 4B,E,H**). This data provided evidence that the lack of MC peptide activities to GPR139 cannot be attributed to loss of peptide sticking to plasticware. In fact, low-binding plasticware was used throughout our studies. Our data clearly demonstrated that ACTH, α-MSH, and β-MSH retained the ability to induce a calcium response in cells co-transfected with GPR139 and MC3R, MC4R, or MC5R (**Figures 4C,F,I**) which was not blocked by a selective GPR139 antagonist (**Figures 6B,E,H**). The ACTH, α-MSH, and β-MSH induced calcium response was also observed in a stable HEK-293 GPR139 cell line transiently expressing MC3R, MC4R, or MC5R further supporting the interaction between GPR139 and MCRs (**Figures 5B–D**).

Interestingly, the interaction we observed between GPR139 and MCRs was specific to HEK-293 cells. We performed co-transfection experiments in CHO-K1 cells transiently expressing MC4R, MC4R + G_α16_ and MC4R + GPR139. Despite the generally lower transfection efficiencies of CHO cells, results from these experiments demonstrated a calcium response to ACTH, α-MSH, and β-MSH in CHO-K1 cells co-expressing MC4R + G_α16_ and a response to the GPR139 agonists in cells co-expressing MC4R + GPR139 (data not shown). One possible explanation for the lack of interaction between MCRs and GPR139 in CHO cells could be that GPR139 has been shown to exist as monomers in HEK-293 cells and dimers in CHO-K1 cells (Süsens et al., 2006). Therefore, MCRs might potentially be forming heterodimers with GPR139 in HEK-293 cells which cannot be assembled in CHO-K1 cells because of GPR139 homodimers.

There are increasing reports in the literature of GPCR homo- and heterodimerizations resulting in cross-talk between GPCR pairs (Fuxe et al., 1998; Naumenko et al., 2014; Borroto-Escuela et al., 2017). One example is the interaction between the melatonin MT_2_ and serotonin 5-HT_2C_ receptors. Kamal et al. (2015) reported that melatonin MT_2_ and serotonin 5-HT_2C_ receptors can form functional heteromers both in transfected HEK-293 cells and in human cortex and hippocampus. Co-expression of MT_2_ (G_i_-coupled) with 5-HT_2C_ (G_q/11_-coupled) receptors in HEK-293 cells increased localization of 5-HT_2C_ receptors to the surface membrane resulting in a potentiation of the serotonin induced response. They further demonstrated melatonin induced inositol phosphate production in a dose-dependent manner in MT_2_/5-HT_2C_ cells which was blocked by the melatonin receptor antagonist S-20928 indicating transactivation of the PLC pathway by activation of the MT_2_ receptor. Conversely, treatment with 5-HT_2C_ neutral antagonists (RS-102221 and SB-242084) had no effect on the melatonin-induced response whereas the inverse agonist SB-206553 blocked the effect suggesting the effect is dependent on the constitutive activity of the 5-HT_2C_ protomer and independent of the occupation of the antagonist binding site. Similarly, in our studies, treatment with the GPR139 antagonist (JNJ-3792165) in MCR/GPR139 expressing cells had no effect on the ACTH, α-MSH and β-MSH-induced calcium response which suggests transactivation of the phospholipase C pathway may also be independent of the GPR139 antagonist binding site.

A range of mechanisms that may be involved in GPCR cross-talk resulting in enhanced intracellular calcium release have been described (Werry et al., 2003). A single mechanism cannot account for all the examples observed to date. In addition, CHO cell populations can be genetically heterogeneous (Davies et al., 2013). The particular CHO line used in our lab may exhibit a slightly different genetic profile from the CHO clonal cell line used by Nøhr et al. (2017b). The upregulation of the MC4R and G_α16_ combined with a more efficient coupling in the population of CHO cells used by Nøhr et al. (2017b) may contribute to the response induced by the MC peptides. Alternatively, the CHO clonal line used by Nøhr et al. (2017b) may be endogenously expressing MC receptors and the recombinantly expressed GPR139 receptor is serving as a signal transducer for the MC peptides. Exploring the underlying mechanism between the MCR and GPR139 are beyond the scope of this study. However, our findings demonstrated that ACTH, α-MSH, and β-MSH are not likely to be physiologically relevant ligands for GPR139. Instead, we hypothesize that an *in vitro* interaction between MCRs and GPR139 resulted in transactivation of the phospholipase C pathway through ACTH, α-MSH, and β-MSH activation of MCRs.

## Author Contributions

DN, CL, TL, and PB: participated in the research design. DN and CK: conducted the experiments. CD: contributed to the new reagents or analytic tools. DN and CK: performed the data analysis. DN and PB: wrote or contributed to the writing of the manuscript.

## Conflict of Interest Statement

DN, CK, CL, CD, TL, and PB are paid employees at Janssen Research and Development, LLC.

## Abbreviations

α-MSH: α melanocyte stimulating hormone
β-MSH: β melanocyte stimulating hormone
ACTH: adrenocorticotropic hormone
cAMP: cyclic adenosine monophosphate
CHO-K1: Chinese hamster ovary
EC_50_: concentration giving 50% of maximal response
EC_100_: concentration giving 100% of maximal response
GPCR: G-protein coupled receptor
GTPγS: guanosine 5′-*O*-(3-thiotriphospate)
HBSS: Hank’s balanced salt solution
HEK-293: human embryonic kidney
L-Phe: L-phenylalanine
L-Trp: L-tryptophan
MC: melanocortin
MCR: melanocortin receptor
MC3R: melanocortin 3 receptor
MC4R: melanocortin 4 receptor
MC5R: melanocortin 5 receptor
PEI: poly(ethyleneimine)
TE: Tris-EDTA.
